# Interactions between rates of temperature change and acclimation affect latitudinal patterns of warming tolerance

**DOI:** 10.1093/conphys/cow053

**Published:** 2016-11-09

**Authors:** Jessica L Allen, Steven L Chown, Charlene Janion-Scheepers, Susana Clusella-Trullas

**Affiliations:** 1 Centre for Invasion Biology, Department of Botany and Zoology, Stellenbosch University, Private Bag X1, Matieland 7602, South Africa; 2 School of Biological Sciences, Monash University, Clayton, VIC 3800, Australia

**Keywords:** Climate change, ectotherms, macrophysiology, phenotypic plasticity, thermal tolerance

## Abstract

Critical thermal limits form an increasing component of the estimation of impacts of global change on ectotherms. Whether any consistent patterns exist in the interactive effects of rates of temperature change (or experimental ramping rates) and acclimation on critical thermal limits and warming tolerance (one way of assessing sensitivity to climate change) is, however, far from clear. Here, we examine the interacting effects of ramping rate and acclimation on the critical thermal maxima (CTmax) and minima (CTmin) and warming tolerance of six species of springtails from sub-tropical, temperate and polar regions. We also provide microhabitat temperatures from 26 sites spanning 5 years in order to benchmark environmentally relevant rates of temperature change. Ramping rate has larger effects than acclimation on CTmax, but the converse is true for CTmin. Responses to rate and acclimation effects are more consistent among species for CTmax than for CTmin. In the latter case, interactions among ramping rate and acclimation are typical of polar species, less marked for temperate ones, and reduced in species from the sub-tropics. Ramping rate and acclimation have substantial effects on estimates of warming tolerance, with the former being more marked. At the fastest ramping rates (>1.0°C/min), tropical species have estimated warming tolerances similar to their temperate counterparts, whereas at slow ramping rates (<0.4°C/min) the warming tolerance is much reduced in tropical species. Rates of temperate change in microhabitats relevant to the springtails are typically <0.05°C/min, with rare maxima of 0.3–0.5°C/min depending on the site. These findings emphasize the need to consider the environmental setting and experimental conditions when assessing species’ vulnerability to climate change using a warming tolerance approach.

## Teaser

Rates of temperature change and thermal acclimation can alter measures of temperature tolerance. Using new experimental data on springtails and data from the literature, we show that these factors interact and have consequences for estimates of organismal vulnerability to climate change at global scales.

## Introduction

Ectotherm physiological performance and fitness are directly affected by temperature. Much attention has been given, therefore, to understanding the ways in which thermal performance curves and their constituent traits vary through space and time (Huey and Kingsolver, 1993; Sunday *et al*., 2011). Recent impetus for understanding the variation in thermal traits has come from the need to forecast the response of populations to changing climates and the ways in which such population dynamics will, in turn, affect species’ vulnerability, geographical range position and size through time (Helmuth *et al*., 2005; Catullo *et al*., 2015; Pacifici *et al*., 2015; Sinclair *et al*., 2016). Among the many significant outcomes of this work, two are notable in the context of environmental change: (i) the finding that thermal tolerance limits can provide accurate means to estimate geographical ranges (Bozinovic *et al*., 2011; Overgaard *et al*., 2014) and warming tolerances (WTs; *sensu*Deutsch et al., 2008) as a proxy for species vulnerability to climate change; and (ii) indications that tropical and sub-tropical species may be substantially more at risk from rising temperatures than their temperate counterparts, although with some complexity about this pattern and the assumptions made to derive it (Deutsch *et al*., 2008; Diamond *et al*., 2012; Hoffmann *et al*. 2013; Sunday *et al*., 2014).

Comparison of critical thermal limits among populations and species has featured prominently in the work underpinning these findings (Clusella-Trullas *et al*., 2011; Duarte *et al*., 2012; Overgaard *et al*., 2014; Buckley *et al*., 2015a; García-Robledo *et al*. 2016). At the same time, two major concerns about such limits and their variation in an environmental change context have arisen. The first concern is the way in which experimental rates of temperature change (hereafter ‘ramping rate’) affect estimates of these limits and the extent to which they show heritable variation (Terblanche *et al*., 2007; Mitchell and Hoffmann, 2010). Several studies, including early investigations, demonstrated that relatively slow ramping rates tend to improve critical thermal limits, probably because of acclimation (Becker and Genoway, 1979; Kelty and Lee, 1999; Powell and Bale, 2004; Sørensen *et al*., 2013). In contrast, other investigations have shown that critical thermal limits are reduced when individuals are exposed to slow ramping rates (Chown *et al*., 2009; Peck *et al*., 2009; Allen *et al*., 2012), probably as a consequence of mounting heat damage over long time periods (Cossins and Bowler, 1987). Much controversy has since arisen about the source of the rate effects and the ways in which they should be treated in an experimental setting (Rezende *et al*., 2011; Terblanche *et al*., 2011; Overgaard *et al*., 2012). Perhaps key among the emerging perspectives is the requirement for an understanding of how rates vary in field conditions (Hoffmann, 2010; Woods *et al*., 2015) and the extent to which the effects of varying rates might, if at all, be consistent among different treatments, taxa and environmental settings (Terblanche *et al*., 2011; Sørensen *et al*., 2013; Rezende *et al*., 2014; Hangartner and Hoffmann, 2015).

The second concern is understanding the way in which phenotypic plasticity varies among upper and lower critical limits and the extent to which such plasticity might affect estimates of the effects of changing environmental temperature on populations (Valladares *et al*., 2014; Catullo *et al*., 2015; Gunderson and Stillman, 2015). Early work suggested that systematic relationships exist between thermal limits, acclimation (as a form of phenotypic plasticity) and geographical range position and/or extent (reviewed by Gaston *et al*., 2009). Later studies have borne out the idea that both basal and plastic variation in upper critical limits tend to be less than that in lower critical limits in ectotherms (Araújo *et al*., 2013; Gunderson and Stillman, 2015). Here too, however, complexity exists about the relationships, depending on the organisms and the environments they inhabit, and the methods adopted for investigation of these effects (Stillman, 2003; Sunday *et al*., 2012; Kaspari *et al*., 2015). Importantly, understanding of how rate variation might affect assessments of plasticity in critical thermal limits, whether these interactions show any consistent variation among taxa owing to phylogenetic or environmental propinquity, and their implications for extinction scenarios under climate change, is poorly developed, despite the importance of comprehending the short-term vs. long-term costs and benefits of physiological plasticity (Chevin *et al*., 2013; Buckley *et al*., 2015b; Catullo *et al*., 2015).

Here, we therefore examine the interacting effects of thermal acclimation (as a form of phenotypic plasticity) and rate of temperature change on thermal tolerance and, in particular, on WT as an estimate of risk from anthropogenic temperature change (Deutsch *et al*., 2008), in six species of springtails (Collembola), from three markedly different environmental settings. The group was selected because of its global importance in soil habitats (Bardgett and van der Putten, 2014), its growing significance from a model organism perspective (Hoskins *et al*., 2015) and because springtails have received little attention compared with insects. Therefore, it offers the opportunity to determine the extent to which variation in rate effects in terrestrial species might be more general.

Specifically, we address four questions. First, what is the extent of variation in rates of temperature change likely to be encountered by springtails in soil environments in distinct latitudes (sub-Antarctic, temperate and sub-tropical)? Second, are the interactions between rate of temperature change and acclimation consistent among upper and lower critical thermal limits and species from markedly different latitudes? Third, do generalizations about the extent of risk from climate change in tropical vs. non-tropical species hold for the springtails investigated here, and to what extent do the acclimation and rate effects alter these estimates, if at all? Finally, are the effects of rate of temperature change on WT discernible at global scales?

## Materials and methods

### Field variation in rates of temperature change

Soil temperature data were recorded hourly just below the soil/litter surface using Thermochron iButtons (DS1922L-F5, 0.5°C resolution; Maxim Integrated, San Jose, CA, USA) along elevational transects: on sub-Antarctic Marion Island (46.89803°S, 37.77475°E; nine sites spanning 7–800 m elevation, 2008–2011; Lee *et al*., 2009), in the temperate Cederberg mountains (Western Cape Province, South Africa, 32.54472°S, 19.41611°E; 11 sites spanning 15–1900 m elevation, 2008–2012; Botes *et al*., 2006) and in the sub-tropical Soutpansberg mountains (Limpopo Province, South Africa, 23.02419°S, 29.42910°E; six sites spanning 800–1700 m elevation, 2009–2012; Munyai and Foord, 2012). Marion Island has a more stable and benign climate than more extreme polar locations on the Antarctic continent or in the Arctic (Smith, 2002; Førland *et al*., 2011) but remains a useful locality reflecting higher latitude environments. Thus, we used the nine sites on the island as exemplars for assessing field variation in rates of change at higher latitudes, although our physiological tests for such areas were undertaken on species from Svalbard in the Arctic (see below). Rate of temperature change was calculated from the change in temperature between consecutive hourly recordings, recognizing that more rapid changes therefore go undetected. Mean rates of temperature change for temperature increases and decreases were evaluated separately, and histograms of rates of change occurring at a single elevation (800–900 m), comparable among transects, produced using R v. 2.14.0, package ggplot2 (Wickham, 2009), were used to illustrate differences among them (Supplementary Table S1 provides full summary statistics for all sites and elevations). A Kruskal–Wallis ANOVA by ranks test, conducted in R, was used to test for significant differences among mean rates of temperature change for temperature increases and decreases both within and among sites.

### Study animals and acclimation conditions

Six Collembola species from three main climatic regions were investigated (Supplementary Table S2). Temperate species originated from the Western Cape Province, South Africa (34°S); polar species were from Svalbard (78°N) but not sub-Antarctic Marion Island because these could not be bred within the time frame of the study; and the sub-tropical species were collected in Mpumalanga Province, South Africa (26°S). All species were collected either by sifting leaf litter and collecting specimens with an aspirator or by extracting Collembola from leaf litter into moist plaster-of-Paris pots using Tullgren funnels. The Svalbard species, *Hypogastrura viatica* and *Xenylla humicola*, were collected in 2010 (78.17451°N, 16.02198°E and 79.07833°N, 13.12527°E, respectively) and maintained in standard Arctic summer conditions (10°C, 24 h light) for at least 3 years prior to the onset of this study. *Folsomia candida* was obtained from a laboratory, mass-bred colony obtained from the ecotoxicology group at Stellenbosch University, originally collected from the Western Cape, and maintained at 15°C (12 h light–12 h dark). *Deuteraphorura* sp. 1 was collected in 2012 from the Tokai Forest Reserve (Cape Town, Western Cape, 34.16555°S, 18.59972°E) and maintained for two generations at 15°C (12 h light–12 h dark). Field collections of the sub-tropical species *Deuteraphoura* sp. 2 and *Hypogastrura cf. assimilis* were undertaken in the Kruger National Park and surrounding areas in the Mpumalanga Province of South Africa (25.18694°S, 31.11222°E and 25.1875°S, 31.82138°E) and were maintained in the laboratory for two generations prior to the study at 20°C (12 h light–12 h dark).

Although the polar species are quite widespread (Fjellberg, 1998; Potapov, 2001), local population adaptation has been found for other polar species from the same family (Birkemoe and Leinaas, 2001; Johnsen, 2014). Some separation in habitats also occurs among the species (Hopkin, 1997; Fjellberg, 1998; Potapov, 2001), but because this is between the soil surface and shallow sub-surface, temperature profiles differ little among them by comparison with global variation (Kearney *et al*., 2014) and the variation among the sites we investigated. In addition, our springtail collection methods were standardized to include only leaf litter and the shallow soil interface.

Mass-bred populations (minimum 150 individuals) were established from the field collections and laboratory colonies. They were housed in 40 ml plastic vials on a damp plaster-of-Paris substrate to prevent desiccation and fed with algae collected from the bark of *Platanus* sp. trees (Hoskins *et al*., 2015). Experiments commenced from the F2 generation of these mass-bred colonies. For the Svalbard species and *F. candida*, some laboratory adaptation may have taken place (Chown and Terblanche, 2007), but we are unable to document the extent of such change if any. Prior to experimental trials, all species were subjected to three temperature treatments (hereafter ‘acclimation treatments’) maintained using controlled-temperature cabinets (MIR-154; SANYO, Osaka, Japan) and verified using Hygrochron iButton loggers (DS 1923-F5). The exposure time for acclimation treatments was 7 days (for rationale, see Weldon *et al*., 2011; Allen *et al*., 2012). The conditions of the control acclimation treatment were set to match those of the standard colony conditions for each latitude group, and the light cycle remained the same for all acclimations. Low and high acclimation temperatures were 10°C below and above standard colony temperatures, respectively, standardizing conditions for measuring the magnitude of acclimation across latitude groups. For the temperate and sub-tropical latitude groups, the high- and low-temperature acclimations fall within the range of the soil temperatures estimated for these areas (Supplementary Table S2). The temperature conditions for the species from Svalbard are higher than mean temperatures for the region but close to summer temperatures when Collembola are active (Coulson, 2013).

### Rate variation and acclimation effects

Springtail critical thermal limits were determined using a double-jacketed aluminium stage connected to a Grant R150 programmable water bath (Grant Instruments Ltd, Cambridge, UK), into which a plastic vial with a damp plaster-of-Paris substrate was fitted. Collembola are highly susceptible to desiccation (Hopkin, 1997), but the plaster-of-Paris substrate provides a humid environment, negating the potentially confounding effects of desiccation on experimental outcomes (for discussion, see Rezende *et al*., 2011). The temperature was monitored on the surface of the plaster using a 40-gauge Type T (copper–constantan) thermocouple attached to a digital thermometer (CHY 507; Thermometer, Taiwan).

The critical thermal maximum (CTmax) and minimum (CTmin) were determined based on the methods of Chown *et al*. (2009) for groups of 10 individuals at a time. More specifically, CTmax was defined as the temperature at which Collembola were incapable of righting themselves. This response was observed while the Collembola lay prone on their side and was typically accompanied by muscular spasms in the legs and extension of the furcula. The CTmin was defined as the temperature at which Collembola were unable to right themselves even when lightly prodded with a fine paintbrush. The loss of righting response is a standard indicator of CTmin and marks the limits of organism functioning in low-temperature conditions, probably associated with impairment of the central nervous system (Hazell and Bale, 2011). This threshold differs from the end of spontaneous movement (e.g. Everatt *et al*., 2013) and lower lethal limits and supercooling points (e.g. Worland and Convey, 2001).

Four ramping rates were used for CTmax (0.5, 0.25, 0.15 and 0.05°C/min) and three for CTmin (0.25, 0.15 and 0.05°C/min), with a starting temperature that matched the colony temperature of the Collembola species being tested and a holding time of 10 min before the ramping commenced. Three replicates of 10 individuals each were undertaken for each treatment, although a few individuals escaped in several of the trials.

For each Collembola species, generalized linear models (Gaussian distribution, identity link function) were used to examine the effects of the rate of temperature change and acclimation on critical thermal limits. Differences between mean CTmax and CTmin measured at the different rates of temperature change were tested for significance using the glht function from R package ‘multcomp’ (Hothorn *et al*., 2008) and the Tukey method. We used linear mixed-effects models fitted by maximum likelihood (ML) estimation [R package lme4 (Bates *et al*., 2015) and lmertest (Kuznetsova *et al*., 2015)] to examine the effects of, and interactions among, rate of temperature change, acclimation treatment and latitude group (i.e. sub-tropical, temperate and polar) on critical thermal limits (fixed effects), across all the Collembola species. Taxonomic identity (family, genera and species) was incorporated as random nested effects to account for phylogenetic relatedness (see e.g. Allgeier *et al*., 2015). This approach was preferred to phylogenetic generalized least-squares methods owing to the number of repeated data within species (several rates and acclimations per species) relative to the total number of species (*n* = 6; Garland *et al*., 2005). Generalized least-squares linear models (R package nlme; Pinheiro *et al*., 2016) were used to test whether a model without the random predictor had a better fit than the linear mixed-effects model (following Zuur *et al*., 2009). Best-fit models were selected using the Akaike information criterion (AIC) using ΔAIC (Burnham and Anderson, 2001). Model validation was carried out using standard approaches (see Supplementary Figs S1–S3). If the final models included random effects, these were fitted and presented using restricted maximum likelihood estimation. The overall effect of rate, acclimation and latitude group in the linear mixed-effects models was tested using the anova function in lme4.

### Warming tolerance

To standardize estimates of warming tolerance and make them comparable to previous approaches that have used interpolated climate data (e.g. Deutsch *et al*., 2008; Hoffmann *et al*., 2013), soil temperature data for all study sites were extracted from the microclimatic data sets of Kearney *et al*. (2014). These data sets provide global estimates of daily hourly microclimatic data based on long-term monthly averages (1960–1990), provided for the central day of each month. Given that the Collembola species used in this study were collected from within the litter layers and soil surface interface, where they spend the majority of their time, we chose to extract temperature data for soil habitats with 50% vegetation shading and at the soil surface. From these data, a mean temperature for the warmest (Tmax) quarter at each site was calculated as the mean of the warmest 3 months of the year.

The WT of each Collembola species was calculated as the difference between CTmax and Tmax (Kingsolver *et al*., 2013; Sunday *et al*., 2014), using the mean CTmax for each rate of temperature change and acclimation treatment. Warming tolerance provides a measure of the relative amount of warming that each species can withstand before reaching critical performance levels (Deutsch *et al*., 2008).

Linear mixed-effects models were used to examine the variation in WT calculated using CTmax from different rates of temperature change and acclimation treatments among latitude groups, and including taxonomic hierarchy (family, genera and species) as random nested effects to account for different phylogenetic relationships across species. The statistical approach, model simplification and validation were carried out as described for CTmax and CTmin analyses above.

The effects of rate of temperature change and absolute latitude on WT were also examined at a global scale by incorporating CTmax data compiled from insect studies in the literature (Supplementary Table S3). A distance-weighted least-squares surface plot was used to illustrate the effects of both rate of temperature change and absolute latitude on WT for multiple species of insects (in Statistica V12.6; StatSoft, Tulsa, OK, USA). For this plot, the mean CTmax of terrestrial insect species was extracted from published papers that incorporated several rates of temperature change (using WebPlotDigitiser V3.7, 12010-2015, Ankit Rohatgi), unless the means were provided in the original papers (Supplementary Table S3). Data from the present study were also included. Two studies incorporated several acclimation treatments. From these studies, temperatures that most closely matched relevant microhabitat temperatures were selected [35 and 20°C acclimation treatments from Terblanche *et al*. (2007) and Chown *et al*. (2009), respectively]. Microsite temperatures or rearing temperatures in the case of laboratory-based colonies reported in studies were used to calculate WT unless these were not available (<5% of cases). For the latter, mean annual temperatures at collection sites were determined from a global temperature data set (http://www.worldclim.org/; Hijmans *et al*., 2005). Latitude was taken directly from the papers or determined based on the reported location of collection (Supplementary Table S4). Where the original collection site could not be determined, the median latitude of the recorded geographical range was obtained by extracting a list of coordinates for the species occurrence records from the Global Biodiversity Information Facility (GBIF; http://www.gbif.org/) using the R package ‘dismo’ (Hijmans *et al*., 2015) or from the literature (in two cases; Supplementary Table S4). A linear mixed-effect model was used to examine the effects of rates of temperature change and latitude on WT and including species as a random effect to account for the non-independence of data within species.

## Results

### Rates of temperature change in the field

**Figure 1:**
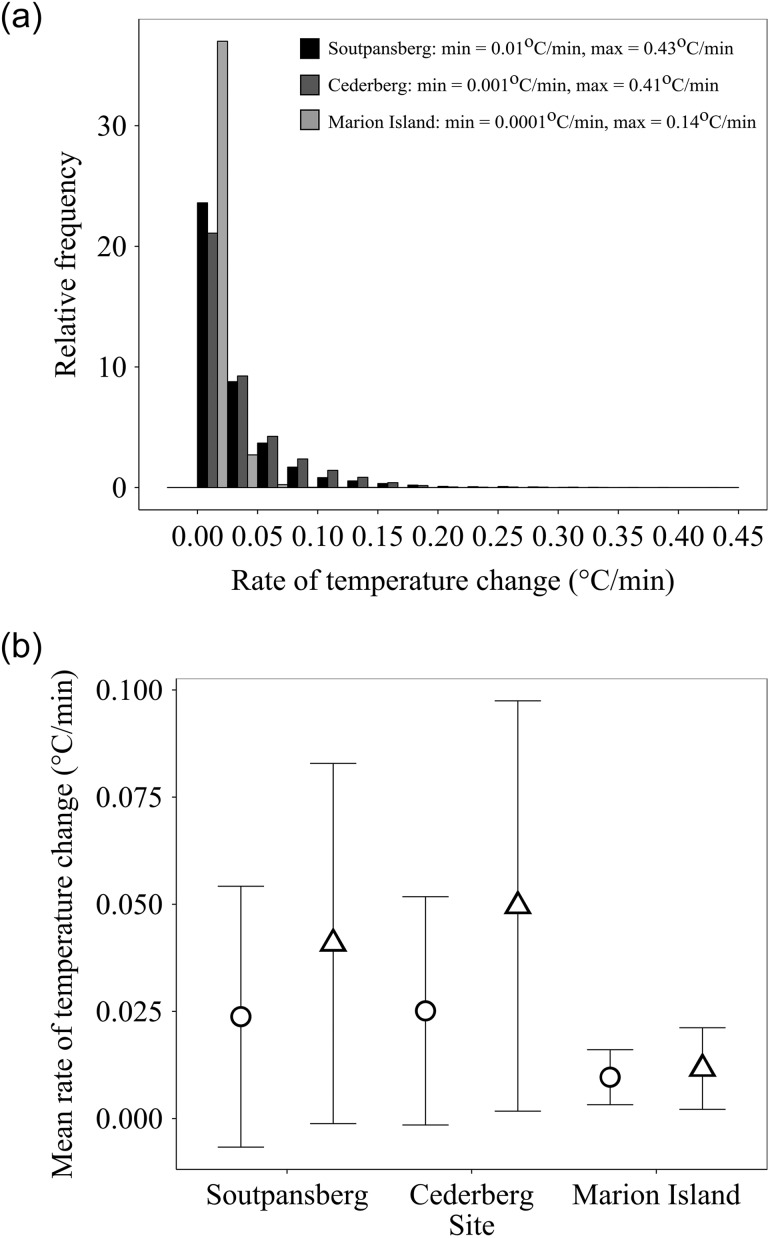
(**a**) Relative frequency of occurrence of rates of temperature change 1 cm below the surface of soils for single sites at comparable altitudes for Marion Island (sub-polar, light grey bars, 800 m above sea level), the Cederberg transect (dark grey, green bars, 900 m above sea level) and the Soutpansberg transect (sub-tropical, black bars, 800 m above sea level). Minimal and maximal rates of temperature change for each site are given in the key. (**b**) Mean rate of temperature change at each site for increasing (open triangles) and decreasing temperatures (open circles). Mean rate of change differed significantly among sites [Kruskal–Wallis H test (2, *n* = 62 066) = 8579.65, *P* < 0.001] and between increasing and decreasing temperatures [Kruskal–Wallis H test (2, *n* = 62 066) = 3544.11, *P* < 0.001]. Vertical bars are standard deviation.

Rates of microclimate temperature change differed among sites and between increasing and declining change trends (Fig. 1). Temperatures in the sub-tropical and temperate areas were characterized by a larger range of rates of change than the sub-polar area. Across all sites (including their constituent altitudinal bands), the fastest rates of temperature change were 0.3°C/min in the sub-polar, 0.4°C/min in the temperate and 0.5°C/min in the sub-tropical sites (Supplementary Table S1). At the sub-polar sites, rates of change <0.01°C/min comprised 76% and rates of change of 0.01–0.05°C/min comprised 23% of the total data set, respectively. For the temperate sites, these values were <0.01°C/min (36%) and 0.01–0.05°C/min (45%), and for the tropical sites <0.01°C/min (42%) and 0.01–0.05°C/min (43%).

### Effects of ramping rate and acclimation on critical thermal limits

**Figure 2:**
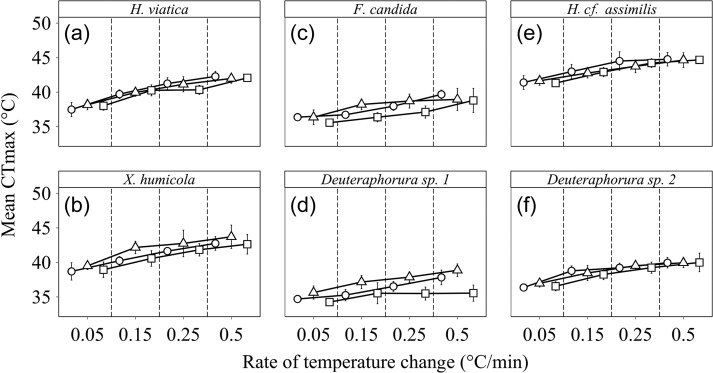
The effect of rate of temperature change and acclimation on the critical thermal maximum (CTmax) of *Hypogastrura viatica* (polar; **a**), *Xenylla humicola* (polar; **b**), *Folsomia candida* (temperate; **c**), *Deuteraphorura* sp. 1 (temperate; **d**), *Hypogastrura cf. assimilis* (sub-tropical; **e**) and *Deuteraphorura* sp. 2 (sub-tropical; **f**). Acclimation effects are: control (open circles, colony temperature), high (open triangles, colony temperature +10°C) and low (open squares, colony temperature −10°C). For each species, *n* = 26–31 at each rate of temperature change for each acclimation treatment.

For all of the species examined, ramping rate effects on CTmax were significant, with the highest mean CTmax measured at the fastest ramping rate (0.5°C/min; Table 1 and Fig. 2). Ramping rates had an effect size of 1.1°C on average (varying from 0.1 to 2.4°C). In contrast, the acclimation effect size was on average half that value (0.5°C), and in some species acclimation had no significant effect on CTmax (Table 1). Although the interactions of ramping rate and acclimation treatment varied among the species (Table 1), the differences were relatively minor (Fig. 2). The small effects of acclimation and the limited differences among latitude groups were also clear in the linear mixed model outcomes (Table 2), especially from the absence of significant three-way interaction terms (Supplementary Table S5). Nonetheless, the basal CTmax values showed substantial interspecific variation (Fig. 2).

**Table 1:** Outcomes of generalized linear models testing for an effect of rate of temperature change and acclimation temperature on the critical thermal maxima of polar, temperate and sub-tropical Collembola species

Latitude group	Species		Estimate	Standard error	*t* value	*P*-value
Polar	*Hypogastrura viatica*	Intercept	37.80	0.15	251.22	**<0.001**
		Rate of temperature change	9.88	0.51	19.25	**<0.001**
		Low-temperature acclimation	0.45	0.21	2.12	**0.035**
		High-temperature acclimation	0.69	0.21	3.30	**0.001**
		Rate*Low	−1.84	0.72	−2.55	**0.011**
		Rate*High	−2.12	0.72	−2.95	**0.003**
	*Xenylla humicola*	Intercept	38.80	0.20	193.54	**<0.001**
		Rate of temperature change	8.67	0.71	12.15	**<0.001**
		Low-temperature acclimation	0.35	0.29	1.23	0.219
		High-temperature acclimation	1.29	0.29	4.48	**<0.001**
		Rate*Low	−0.93	1.00	−0.93	0.354
		Rate*High	−0.45	1.02	−0.44	0.659
Temperate	*Folsomia candida*	Intercept	35.88	0.16	223.32	**<0.001**
		Rate of temperature change	7.64	0.55	13.82	**<0.001**
		Low-temperature acclimation	−0.60	0.23	−2.62	**0.008**
		High-temperature acclimation	1.02	0.23	4.50	**<0.001**
		Rate*Low	−0.53	0.78	−0.68	0.497
		Rate*High	−2.80	0.78	−3.58	**<0.001**
	*Deuteraphorura* sp. 1	Intercept	34.38	0.16	219.53	**<0.001**
		Rate of temperature change	7.09	0.54	13.25	**<0.001**
		Low-temperature acclimation	0.34	0.23	1.50	0.135
		High-temperature acclimation	1.40	0.22	6.29	**<0.001**
		Rate*Low	−5.00	0.77	−6.51	**<0.001**
		Rate*High	−0.44	0.76	−0.58	0.562
Sub-tropical	*Hypogastrura cf. assimilis*	Intercept	41.73	0.18	226.49	**<0.001**
		Rate of temperature change	7.18	0.65	11.00	**<0.001**
		Low-temperature acclimation	−0.10	0.26	−0.37	0.711
		High-temperature acclimation	−0.16	0.26	−0.62	0.539
		Rate*Low	−0.22	0.90	−0.25	0.805
		Rate*High	0.13	0.93	0.14	0.888
	*Deuteraphorura* sp. 2	Intercept	37.00	0.16	233.12	**<0.001**
		Rate of temperature change	6.75	0.54	12.44	**<0.001**
		Low-temperature acclimation	−0.17	0.22	−0.75	0.453
		High-temperature acclimation	0.34	0.22	1.50	0.136
		Rate*Low	0.31	0.76	0.41	0.680
		Rate*High	−0.77	0.77	−1.00	0.316

Significant results are shown in bold. Sample sizes varied from 26 to 31 individuals per rate and acclimation treatment.

**Table 2:** Results of ANOVA of the linear mixed-effects models testing for the effects of rates of temperature change (in degrees Celsius per minute), acclimation treatment (in degrees Celsius) and latitude group on CTmax, CTmin and warming tolerance

Response variable	d.f.	*F*-value
CTmax		
Rate	1	2553.0***
Acclimation	2	39.35***
Latitude group	2	2.09
Rate*Acclimation	1	9.60***
Rate*Latitude group	2	27.54***
Acclimation*Latitude group	2	8.35***
Rate*Acclimation*Latitude group	2	2.90*
CTmin		
Rate	1	22.61***
Acclimation	2	202.13***
Latitude group	2	3.06
Rate*Acclimation	1	8.65***
Rate*Latitude group	2	22.50***
Acclimation*Latitude group	2	17.69***
Rate*Acclimation*Latitude group	2	3.93*
Warming tolerance		
Rate	1	2922.0***
Acclimation	2	38.95***
Latitude group	2	100.95*
Rate*Acclimation	2	9.46***
Rate*Latitude group	2	26.86***
Acclimation*Latitude group	4	8.21***
Rate*Acclimation*Latitude group	4	2.87*

Model estimates and SE are presented in Supplementary Table S5. CTmax, critical thermal maximum; CTmin, critical thermal minimum. For CTmax, the denominator d.f. = 2130; for CTmin, d.f. = 1571; for warming tolerance, d.f. = 2110, except for latitude group d.f. = 3 in all models. *P < 0.05, **P < 0.001 and ***P < 0.0001.

**Figure 3:**
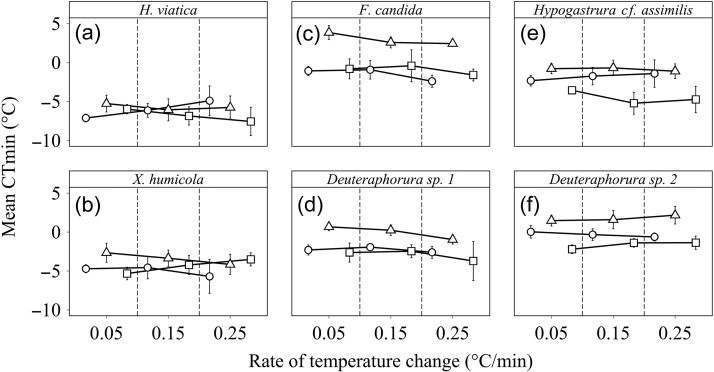
The effect of rate of temperature change and acclimation on the critical thermal minimum (CTmin) of *Hypogastrura viatica* (polar; **a**), *Xenylla humicola* (polar; **b**), *Folsomia candida* (temperate; **c**), *Deuteraphorura* sp. 1 (temperate; **d**), *Hypogastrura cf. assimilis* (sub-tropical; **e**) and *Deuteraphorura* sp. 2 (sub-tropical; **f**). Acclimation effects are: control (open circles, colony temperature), high (open triangles,  colony temperature +10°C) and low (open squares, colony temperature −10°C). For each species, *n* = 26–31 at each rate of temperature change for each acclimation treatment.

The responses of CTmin to the combined effects of ramping rate and acclimation treatment were more variable within and among species and latitude groups than those of CTmax (Tables 2 and 3 and Fig. 3). Two differences between the responses of CTmax and CTmin to acclimation and ramping rate are notable. First, for CTmin, acclimation had a greater effect than ramping rate, although both were often significant. Second, the effect of ramping rate on CTmin varied with acclimation treatment, both in direction and in the size of the effect, depending on the species. However, these differences were less notable in mixed-effects models (Supplementary Table S5). For the polar species, the response of mean CTmin to the rate of temperature change was significantly affected by acclimation treatment, and interactions between ramping rate and acclimation were all significant (Fig. 3 and Table 3). In the temperate species, mean CTmin generally increased as ramping rate declined, and mean CTmin following high-temperature acclimation was significantly higher (by ~1.6–4.2°C) across all rates of change compared with the other acclimation treatments (Fig. 3 and Table 3). In both sub-tropical species, high- and low-temperature acclimations resulted in higher and lower mean CTmin, respectively, compared with the control acclimation treatment across all ramping rates (Fig. 3 and Table 3).

**Table 3:** Outcomes of generalized linear models testing for an effect of rate of temperature change and acclimation temperature on critical thermal minima of polar, temperate and sub-tropical Collembola species

Latitude group	Species		Estimate	Standard error	*t* value	Pr(>|*t*|)
Polar	*Hypogastrura viatica*	Intercept	−7.72	0.30	−26.16	**<0.001**
		Rate of temperature change	11.13	1.70	6.55	**<0.001**
		Low-temperature acclimation	2.12	0.41	5.14	**<0.001**
		High-temperature acclimation	2.37	0.42	5.65	**<0.001**
		Rate*Low	−19.08	2.39	−7.98	**<0.001**
		Rate*High	−13.49	2.41	−5.59	**<0.001**
	*Xenylla humicola*	Intercept	−4.27	0.27	−15.80	**<0.001**
		Rate of temperature change	−4.83	1.61	−3.01	**0.003**
		Low-temperature acclimation	−1.45	0.39	−3.75	**<0.001**
		High-temperature acclimation	2.00	0.40	5.04	**<0.001**
		Rate*Low	13.94	2.29	6.08	**<0.001**
		Rate*High	−2.64	2.33	−1.13	0.259
Temperate	*Folsomia candida*	Intercept	−0.46	0.25	−1.86	0.064
		Rate of temperature change	−6.63	1.45	−4.58	**<0.001**
		Low-temperature acclimation	0.11	0.35	0.32	0.748
		High-temperature acclimation	4.50	0.35	12.93	**<0.001**
		Rate*Low	2.73	2.04	1.34	0.182
		Rate*High	−0.56	2.06	−0.27	0.787
	*Deuteraphorura* sp. 1	Intercept	−2.05	0.25	−8.23	**<0.001**
		Rate of temperature change	−1.60	1.42	−1.13	0.260
		Low-temperature acclimation	−0.08	0.35	−0.23	0.821
		High-temperature acclimation	3.26	0.35	9.37	**<0.001**
		Rate*Low	−3.69	2.03	−1.81	**0.071**
		Rate*High	−6.56	2.01	−3.27	**0.001**
Sub-tropical	*Hypogastrura cf. assimilis*	Intercept	−2.50	0.27	−9.23	**<0.001**
		Rate of temperature change	4.55	1.59	2.87	**0.004**
		Low-temperature acclimation	−1.14	0.39	−2.89	**0.004**
		High-temperature acclimation	1.89	0.38	5.02	**<0.001**
		Rate*Low	−10.43	2.31	−4.52	**<0.001**
		Rate*High	−6.17	2.21	−2.80	**0.006**
	*Deuteraphorura* sp. 2	Intercept	0.18	0.18	1.03	0.306
		Rate of temperature change	−3.28	1.05	−3.13	**0.002**
		Low-temperature acclimation	−2.47	0.25	−9.74	**<0.001**
		High-temperature acclimation	1.04	0.25	4.14	**<0.001**
		Rate*Low	7.48	1.49	5.03	**<0.001**
		Rate*High	6.75	1.48	4.55	**<0.001**

Significant results are shown in bold text. Sample sizes varied from 26 to 31 individuals per rate and acclimation treatment.

### Warming tolerance

**Figure 4:**
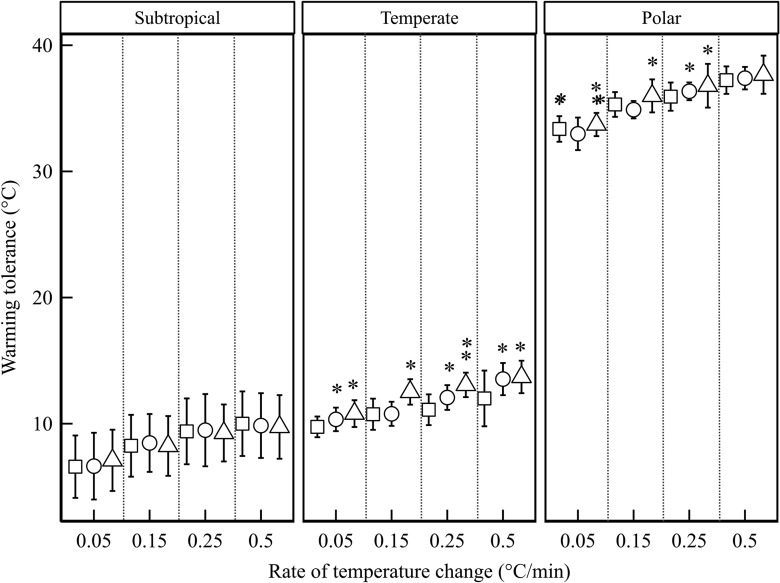
(**a**) Mean (±SD) of springtail warming tolerance defined as CTmax – Tmax (see Materials and methods) for latitude groups, measured at different rates of temperature change under low (open squares, colony temperature −10°C), control (open circles, colony temperature) and high (open triangles, colony temperature +10°C) acclimation treatments. Lack of symbol or same number of asterisks denotes no significant differences (*P* > 0.05) in warming tolerance among acclimations within each rate of temperature change.

**Figure 5:**
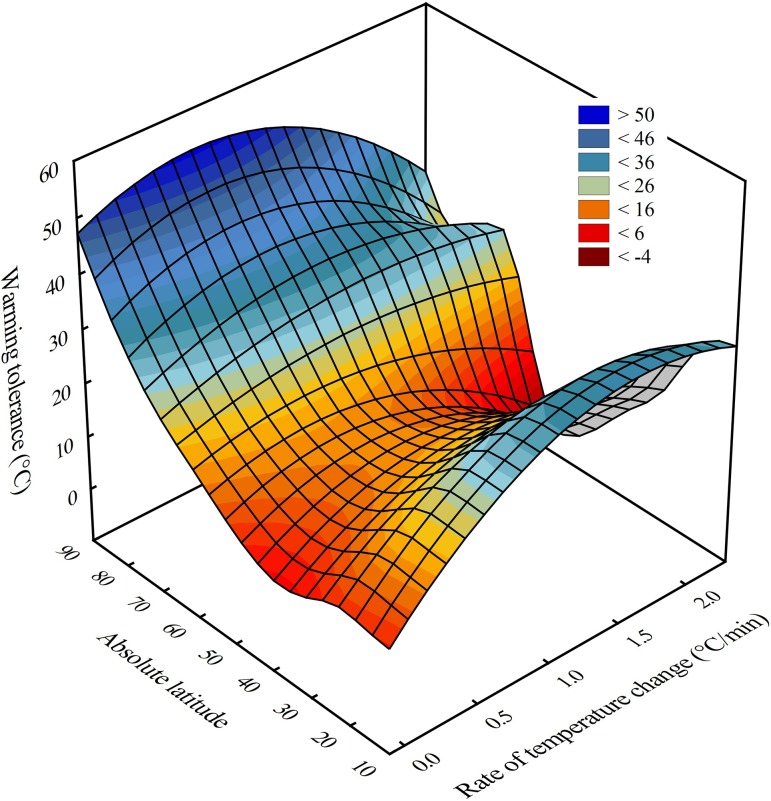
Distance-weighted least-squares surface plot illustrating the effects of rate of temperature change on warming tolerance of terrestrial insects and springtails at global scales. Warming tolerance estimates were calculated from CTmax data compiled from the literature (see Materials and methods).

Given that ramping rate and acclimation treatment had significant effects on the CTmax values, they necessarily influenced estimates of warming tolerance, although more so for ramping rate than for acclimation (Table 2; see Supplementary Table S6 for outcomes of model selection). Generally, warming tolerance increased significantly with increasing ramping rate (Fig. 4). Acclimation treatment had little effect on the warming tolerances of sub-tropical species, whereas acclimation effects varied among the temperate and polar species (Fig. 4). Polar species had a significantly higher warming tolerance (by ~22.6–27.9°C) than temperate and sub-tropical species (Fig. 4 and Table 2). The latter did not differ when considering all ramping rates (estimate = −0.90, standard error = 1.45, d.f. = 6, *t* = −2.00, *P* = 0.092) but did so when 0.05°C/min alone was considered, with tropical species having the lowest warming tolerance (estimate = −3.79, standard error = 1.48, d.f. = 6, *t* = −2.56, *P* = 0.043). For warming tolerance estimates compiled from the literature, both rate (*F*_1,50_ = 9.29, *P* = 0.0037) and latitude (*F*_1,50_ = 14.26, *P* = 0.0004) affected warming tolerance, with a marginal significant interaction of rate and latitude (*F*_1,50_ = 3.99, *P* = 0.051; Fig. 5).

## Discussion

Several key results emerge from this investigation of the effects of ramping rate and acclimation on springtail thermal tolerances and the rates of temperature change these species are likely to encounter in the field. Most significantly, ramping rate affected CTmax more than CTmin, having substantial consequences for estimates of warming tolerance. Moreover, experimental rates also interacted with acclimation effects on critical limits, but with an indication of emerging generality to these effects, rather than simply an unpredictable or haphazard outcome. Given the growing exploration of CTmax and warming tolerance in the context of both fundamental physiological ecology and the conservation implications of climate change, and a broadening in the range of experimental ramping rates used (for discussions and/or examples, see Terblanche *et al*. 2011; Sunday *et al*., 2012, 2014; Baudier *et al*., 2015; Kaspari *et al*., 2015; García-Robledo *et al*., 2016), these results have important implications for future work. We explore these implications in more detail here.

The CTmax showed generally consistent interspecific patterns of a much larger effect of ramping rate than of acclimation. Limited acclimation responses are characteristic of upper thermal limits, including CTmax (Hoffmann *et al*., 2013; Gunderson and Stillman, 2015). The substantial effects of ramping rate have also been recorded in several investigations (e.g. Terblanche *et al*., 2007; Ribeiro *et al*., 2012). In consequence, these outcomes support the general view that variation in critical thermal maxima is relatively constrained (e.g. Hoffmann *et al*., 2013). Nonetheless, notable features of the six species investigated here are the CTmax variation of >6°C in the same ramping and acclimation conditions and the fact that faster ramping rates did not necessarily lead to higher CTmax values (see e.g. Fig. 2d). Thus, variation in upper relative to lower thermal limits is constrained, but there is some scope for both evolutionary and shorter-term variation in upper thermal tolerance (see also Chown and Terblanche, 2007; Araújo *et al*., 2013; Hangartner and Hoffmann, 2015). Moreover, assumptions that a straightforward time effect accounts for CTmax variation among different ramping rates (see the formulation in Rezende *et al*., 2014) require further empirical evaluation.

In the case of CTmin, the larger effects of acclimation than of ramping rate are in keeping with previous investigations (e.g. Chown and Terblanche, 2007). More notable is the interspecific variation in the way that ramping rate affects assessments of the outcomes of acclimation. Such interactions have been documented previously (e.g. Chown *et al*., 2009), but no studies have suggested that there might be a consistent latitudinal effect. Here, for the sub-tropical species, rate tended not to have a large effect on the sign of the effect of acclimation, as has also been found for the sub-tropical *Drosophila melanogaster* (Chown *et al*., 2009; and see Keller, 2007 for discussion of the origin of this species). The interaction effects were somewhat more pronounced for the temperate springtail species, as was also the case for the more temperate ant *Linepithema humile* (Chown *et al*., 2009). In the polar species, however, ramping rate effects entirely altered assessments of the outcomes of acclimation. In some cases, acclimation effects were as might be expected. For example, acclimation at low/high temperatures lowered/raised CTmin relative to controls using slow ramping rates in *X. humicola*. But in others, this was not the case; using fast ramping rates the situation in *X. humicola* was quite different, with the low-temperature acclimation treatment having the highest CTmin. In *H. viatica*, almost the converse was found. Clearly, much needs to be done to understand the way in which stress intensity and physiological responses vary among different species depending on their history and on the environment in which they have evolved. Importantly, however, the data here suggest that the effects are not haphazardly distributed among species, but rather that a consistent effect might be found among species from different environmental settings (see also Gaston *et al*., 2009). Despite the need to investigate additional species across latitudes to increase predictive power, such a consistent environmental effect, if general, would constitute an additional macrophysiological generalization, helpful for understanding and forecasting ectotherm responses to environmental variation (see Chown and Gaston, 2016).

The interactive effects of ramping rate and acclimation on critical limits mean that they will also affect estimates of thermal tolerance range, frequently used along with critical limits to understand the mechanistic basis of geographical range variation (Bozinovic *et al*., 2011; Sinclair *et al*., 2016). An important consideration, therefore, is what rate is appropriate for assessments of critical thermal limits? The question goes back to the earliest studies, which settled on a rate of ~0.34°C/min as a compromise to avoid the artefacts of thermal inertia at high rates and physiological responses at low rates (Becker and Genoway, 1979), and which was subsequently widely adopted (see e.g. Dallas and Rivers-Moore, 2012). More recent work has suggested that environmentally relevant rates of change should be adopted or at least considered (e.g. Terblanche *et al*., 2007, 2011) and that, typically, these are slow, although with much variation around them. Our microclimate data from three very different sites support the idea that rates of temperature change are often <0.01°C/min but at times do reach values as high as 0.5°C/min. In consequence, for studies that seek single estimates of critical thermal limits, high rates of change, such as those >0.5°C/min, should probably be avoided, unless specific circumstances can be demonstrated for which they might be applicable. Such consideration is important given that a wide range of studies are now routinely investigating critical thermal limits (e.g. Baudier *et al*., 2015; Kaspari *et al*., 2015; Verble-Pearson *et al*., 2015; García-Robledo *et al*., 2016). A contrary view is that both temperature and time should be investigated fully owing to the significance of a third parameter, the sensitivity to temperature change (Rezende *et al*., 2014). The significance of this parameter in a critical thermal limit context is yet to be established fully, especially as it predicts CTmin values for insects as low as −107°C (Rezende *et al*., 2014), which is nearly double the lowest average freezing point ever recorded for an insect (Sinclair, 1999).

From the perspective of population sensitivity to global climate change, it is clear that rates of experimental temperature change have a considerable influence on estimates of warming tolerance and its latitudinal variation (Fig. 5). At the fastest rates of change, both tropical and high-latitude species have the highest warming tolerance, with mid-latitude species showing much lower warming tolerance. In contrast, at slower rates of temperature change, the outcomes are much more in keeping with the original studies (Deutsch *et al*., 2008; Huey *et al*., 2009), where warming tolerance is lowest for tropical and sub-tropical species and subsequently increases. Thus, for both springtails and insects, warming tolerance at typically recorded environmental rates of change is lowest for the more tropical species, although noting that these estimates need to factor in both behavioural responses and life stages that may show the greatest thermal sensitivity (Sunday *et al*., 2014; Levy *et al*., 2015).

The effects of the ramping rate on estimates of warming tolerance highlight the importance of subtleties of the environmental setting when estimating population risk. Warming tolerance will vary depending on whether macroclimate or microclimatic conditions are being used for the calculations, with some investigations showing greater WT in microclimate conditions and others revealing the converse (see Andrew *et al*., 2013; Sunday *et al*., 2014; Pincebourde and Casas, 2015). Experimental rates of change routinely experienced by organisms are also likely to influence estimates of critical thermal maximum, which in turn means that their effects will affect estimates of warming tolerance. Moreover, the interaction is complicated if the most stressful periods are those likely also to be associated with the highest rates of environmental temperature change (Helmuth *et al*., 2005). In consequence, understanding the rates of change likely to be experienced by organisms in a given setting and knowing the timing of the exposure are important when determining the extent of risk to populations under climate change and designing mitigation strategies for conserving diversity in the future (see also Dowd *et al*., 2015).

In conclusion, this study has shown that consistent patterns in the interactive effects of rate of temperature change and acclimation on critical thermal limits are discernible among latitudinal groups of springtail species. More generally, it provides further support for the findings of limited acclimation effects in upper relative to lower critical limits and the elevated risks that tropical and sub-tropical species are facing from ongoing climate change. Crucially, it highlights the importance of considering environmental rates of temperature change and microclimate information in the assessment of warming tolerance as a proxy of species’ vulnerability to climate change.

## Supplementary material

Supplementary material is available at *Conservation Physiology* online.

## Supplementary Material

Supplementary Data
