# Supplementary material for: Interactions between rates of temperature change and acclimation affect latitudinal patterns of warming tolerance
Source: Conserv Physiol. 2016 Nov 9;4(1):cow053. doi: 10.1093/conphys/cow053 (PMC5142048; doi:10.1093/conphys/cow053)
Supplement: Supplementary Data [file cow053_suppldata_final2.docx]

**Supplementary data**

**Table S1:** Descriptive statistics for rates of temperature change (°C/min) occurring in polar (Marion Island), temperate (Cederberg) and subtropical (Soutpansberg) soils at a range of altitudes.

| Transect | Altitude (m a.s.l.) | Mean | Median | Mode | Minimum | Maximum | Range |
| --- | --- | --- | --- | --- | --- | --- | --- |
| Marion Island | 0 | 0.007 | 0.008 | 0.008 | 0.000 | 0.110 | 0.109 |
| Marion Island | 100 | 0.010 | 0.008 | 0.008 | 0.001 | 0.161 | 0.160 |
| Marion Island | 200 | 0.010 | 0.008 | 0.008 | 0.000 | 0.127 | 0.127 |
| Marion Island | 300 | 0.012 | 0.008 | 0.008 | 0.001 | 0.194 | 0.193 |
| Marion Island | 400 | 0.013 | 0.008 | 0.008 | 0.001 | 0.186 | 0.185 |
| Marion Island | 500 | 0.014 | 0.008 | 0.008 | 0.000 | 0.310 | 0.310 |
| Marion Island | 600 | 0.012 | 0.008 | 0.008 | 0.001 | 0.167 | 0.166 |
| Marion Island | 700 | 0.011 | 0.008 | 0.008 | 0.001 | 0.142 | 0.142 |
| Marion Island | 800 | 0.011 | 0.008 | 0.008 | 0.000 | 0.144 | 0.144 |
| Cederberg | 0 | 0.028 | 0.017 | 0.008 | 0.008 | 0.199 | 0.191 |
| Cederberg | 200 | 0.034 | 0.017 | 0.008 | 0.008 | 0.258 | 0.250 |
| Cederberg | 300 | 0.034 | 0.017 | 0.008 | 0.008 | 0.314 | 0.306 |
| Cederberg | 500 | 0.038 | 0.025 | 0.008 | 0.008 | 0.317 | 0.308 |
| Cederberg | 700 | 0.037 | 0.017 | 0.008 | 0.007 | 0.366 | 0.359 |
| Cederberg | 900 | 0.038 | 0.025 | 0.008 | 0.008 | 0.431 | 0.422 |
| Cederberg | 1100 | 0.027 | 0.017 | 0.008 | 0.008 | 0.398 | 0.390 |
| Cederberg | 1300 | 0.026 | 0.017 | 0.008 | 0.000 | 0.333 | 0.333 |
| Cederberg | 1500 | 0.034 | 0.017 | 0.008 | 0.001 | 0.373 | 0.372 |
| Cederberg | 1700 | 0.037 | 0.017 | 0.008 | 0.008 | 0.384 | 0.375 |
| Cederberg | 1900 | 0.028 | 0.017 | 0.008 | 0.000 | 0.350 | 0.350 |
| Soutpansberg | 800 | 0.031 | 0.017 | 0.008 | 0.008 | 0.425 | 0.417 |
| Soutpansberg | 1000 | 0.032 | 0.017 | 0.008 | 0.008 | 0.389 | 0.381 |
| Soutpansberg | 1200 | 0.038 | 0.017 | 0.008 | 0.008 | 0.523 | 0.515 |
| Soutpansberg | 1400 | 0.032 | 0.017 | 0.008 | 0.008 | 0.437 | 0.429 |
| Soutpansberg | 1600 | 0.040 | 0.017 | 0.008 | 0.008 | 0.365 | 0.357 |
| Soutpansberg | 1700 | 0.035 | 0.025 | 0.008 | 0.008 | 0.425 | 0.417 |

Table S2: Acclimation treatments for Collembola species based on latitudinal group. Polar species (*Hypogastrura viatica*, *Xenylla humicola*) originate from Svalbard (Norway), temperate species (*Deuteraphorura* sp.1, *Folsomia candida*), from the Western Cape Province (South Africa) and the subtropical species (*Deuteraphoura* sp. 2, *Hypogastrura cf. assimilis*) from the Mpumalanga Province (South Africa). Soil microclimate temperatures were calculated from extracted data for 1 cm soil depth under 50% vegetation cover from Kearney *et al.* (2014). Mean summer (Tmax), winter (Tmin) and annual (Tann) temperatures are provided.

| Latitude group | Acclimation temperatures (°C) | | | Light cycle | Soil microclimate temperatures (°C) | | |
| --- | --- | --- | --- | --- | --- | --- | --- |
|  | Control | Low | High |  | Tmax | Tmin | Tann |
| Polar | 10 | 0 | 20 | 24 hour Light | 5.1 | -20.1 | -8.7 |
| Temperate | 15 | 5 | 25 | 12h:12h, L: D | 25.2 | 11.2 | 18.3 |
| Subtropical | 20 | 10 | 30 | 12h:12h, L: D | 32.3 | 17.6 | 25.7 |

Reference: Kearney MR, Isaac AP, Porter WP (2014) microclim: Global estimates of hourly microclimate based on long-term monthly climate averages. *Scientific Data* 1: 140006 doi: 10.1038/sdata.2014.6

**Table S3:** The effects of rate of temperature change and/or acclimation on critical thermal limits (CTLs) from empirical studies of terrestrial invertebrates. Rate effect size is the difference between the CTLs measured at the lowest and highest rates of temperature change. Only a single study (Chown *et al.,* 2009) incorporates both rate and acclimation effects and the interaction between rate and acclimation effects were only significant for *Linepithema humile*.

| Species | Rate effect | Rates used (°/min) | Rate effect size (°C) | Rate vs. Acclimation | Reference |
| --- | --- | --- | --- | --- | --- |
| *Drosophila melanogaster* |  | 0.05, 0.1, 0.25, 0.5 |  |  | Chown *et al.,* 2009 |
| CTmax | Positive |  | 1.2 | Rate > Acclimation |  |
| CTmin | Positive |  | 0.5 | Rate < Acclimation |  |
| *Linepithema humile* |  | 0.05, 0.1, 0.25, 0.5 |  |  |  |
| CTmax | Positive |  | 6.8 | Rate > Acclimation |  |
| CTmin | Negative |  | 2.1 | Rate > Acclimation |  |
| *Glossina pallidipes* |  | 0.06, 0.12, 0.25 |  |  | Terblanche *et al.,* 2007 |
| CTmax | Positive |  | 5.0 |  |  |
| CTmin | Negative |  | 10 |  |  |
| *Glossina pallidipes* |  | 0.06, 0.12, 0.25 |  |  | Terblanche *et al.,* 2008 |
| CTmax | Positive |  | 3.6 |  |  |
| CTmin | Negative |  | 9.1 |  |  |
| *Ceratitis capitata* |  | 0.06, 0.12, 0.25 |  |  | Nyamukondiwa and Terblanche, 2010 |
| CTmax | Negative |  | 0.3 |  |  |
| CTmin | Positive |  | 0.7 |  |  |
| *Ceratitis rosa* |  | 0.06, 0.12, 0.25 |  |  |  |
| CTmax | Negative |  | 0.5 |  |  |
| CTmin | Positive |  | 0.6 |  |  |
| *Cydia pomonella* |  | 0.06, 0.12, 0.25 |  |  | Chidawanyika and Terblanche, 2011 |
| CTmax | Negative |  | 1.5 |  |  |
| CTmin | Positive |  | 0.4 |  |  |
| *Tenebrio molitor* |  | 0.05, 0.15, 0.25, 0.5 |  |  | Allen *et al.,* 2012 |
| CTmax | Positive* |  | 2.0 |  |  |
| CTmin | Positive |  | 3.0 |  |  |
| *Cyrtobagous salviniae* |  | 0.05, 0.15, 0.25, 0.5 |  |  |  |
| CTmax | Positive |  | 6.0 |  |  |
| CTmin | Negative |  | 4.0 |  |  |
| *Drosophila melanogaster* |  | 0.06, 0.1 |  |  | Overgaard *et al.,* 2012 |
| CTmax | Positive |  | 0.7 |  |  |
| *Atta sexdens rubropulosa* |  | 0.16, 0.18, 0.2, 0.22, 0.25, 0.29, 0.33, 0.4, 0.5, 0.66, 1, 2 |  |  | Ribeiro *et al.,* 2012 |
| CTmax | Positive |  | 3.0 |  |  |

| *Initially positive, negative between 0.25 and 0.5°C/min |
| --- |

**Table S4:** Sources of latitude for the terrestrial arthropod species for which data on rate effects on critical thermal limits are available. Where specific collection sites were not given in published papers, the median of the known latitudinal range was obtained from the Global Biodiversity Information Facility (GBIF, www.gbif.org).

| Species | Absolute latitude (°) | Reference | Latitude obtained |
| --- | --- | --- | --- |
| *Atta sexdens* | 23.561551 | Ribeiro *et al.,* 2012 | Not specified in paper, probably Sao Paulo. GBIF median latitude |
| *Hypogastrura* *cf*. *assimilis* | 26 | This study | Collection site known from JLA |
| *Ceratitis capitata* | 33.932804 | Nyamukondiwa and Terblanche, 2010 | Starter colonies collected from Stellenbosch and surroundings. |
| *Cydia pomonella* | 33.932804 | Chidawanyika and Terblanche, 2011 | Original colony established in Stellenbosch |
| *Ceratitis rosa* | 33.932804 | Nyamukondiwa and Terblanche, 2010 | Starter colonies collected from Stellenbosch and surroundings. |
| *Cyrtobagous salviniae* | 26.303181 | Allen *et al.,* 2012 | Using collection site of Holotype (Calder and Sands 1985) |
| *Drosophila* *melanogaster* | 23 | Chown *et al.,* 2009 | Keller 2007 |
| *Drosophila* *melanogaster* | 23 | Overgaard *et al.,* 2012 | Keller 2007 |
| *Deuteraphorura* sp. 1 | 34 | This study | Collection site known from JLA |
| *Deuteraphorura* sp. 2 | 26 | This study | Collection site known from JLA |
| *Folsomia* *candida* | 52.15003 |  | GBIF median latitude |
| *Glossina pallidipes* | 13.166902 | Terblanche *et al.,* 2007 | Collection site specified in paper |
| *Hypogastrura viatica* | 78.17451 | This study | Collection site from author CJS |
| *Linepithema humile* | 33.916689 | Chown *et al.,* 2009 | Collection site specified in paper |
| *Tenebrio molitor* | 57.2671 | Allen *et al.,* 2012 | GBIF median latitude |
| *Xenylla humicola* | 79.0783 | This study | Collection site from author CJS |

**References**

Allen JL, Clusella-Trullas S, Chown SL (2012) The effects of acclimation and rates of temperature change on critical thermal limits in *Tenebrio molitor* (Tenebrionidae) and *Cyrtobagous salviniae* (Curculionidae). *J Insect Physiol* 58**:** 669-678.

Calder AA, Sands DPA (1985) A new Brazilian *Cyrtobagous* Hustache (Coleoptera: Curculionidae) introduced into Australia to control *Salvinia. J Aust Entomol Soc* 24: 57-64.

Chidawanyika F, Terblanche JS (2011) Rapid thermal responses and thermal tolerance in adult codling moth *Cydia pomonella* (Lepidoptera: Tortricidae). *J Insect Physiol* 57: 108-117.

Chown SL, Jumbam KR, Sørensen JG, Terblanche JS (2009) Phenotypic variance, plasticity and heritability estimates of critical thermal limits depend on methodological context. *Funct Ecol* 23**:** 133-140.

Keller A (2007) *Drosophila melanogaster*'s history as a human commensal. *Curr Biol* 17: R77-R81.

Nyamukondiwa C, Terblanche JS (2010) Within-generation variation of critical thermal limits in adult Mediterranean and Natal fruit flies *Ceratitis capitata* and *Ceratitis rosa*: thermal history affects short-term responses to temperature. *Physiol Entomol* 35: 255-264.

Overgaard J, Kristensen TN, Sørensen JG (2012) Validity of thermal ramping assays used to assess thermal tolerance in arthropods. *PLoS ONE* 7: e32758.

Ribeiro PL, Camacho A, Navas CA (2012) Considerations for assessing maximum critical temperatures in small ectothermic animals: insights from leaf-cutting ants. *PLoS One* 7: e32083.

Terblanche JS, Deere JA, Clusella-Trullas S, Janion C, Chown SL (2007) Critical thermal limits depend on methodological context. *Proc R Soc B* 27: 2935-2942.

Terblanche JS, Clusella-Trullas S, Deere JA, Chown SL (2008) Thermal tolerance in a south-east African population of the tsetse fly *Glossina pallidipes* (Diptera, Glossinidae): Implications for forecasting climate change impacts. *J Insect Physiol* 54: 114-127.

**Table S5:** Outcomes of best fit Linear Mixed Models (LMMs) fit by Maximum Likelihood (ML) examining the interactions between rate of temperature change, acclimation treatment and latitude group on CTmax, CTmin and Warming tolerance. LMM models include rate, acclimation and latitude group as fixed effects and species as a random effect. Model validations supplied in Appendix E. Significant results shown in bold.

| LMM fit by REML | Estimate | SE | df | t value | p |  |
| --- | --- | --- | --- | --- | --- | --- |
| CTmax: *lmer(CTmax~Rate*Acc*LatGrp+(1\|Fam:Gen:Sp))* |  |  |  |  |  |  |
| Intercept | 38.29 | 1.45 | 3 | 26.46 | **< 0.001** |  |
| Rate of temperature change | 9.32 | 0.41 | 2129.8 | 22.47 | **< 0.0001** |  |
| High acclimation treatment | 0.99 | 0.17 | 2129.8 | 5.90 | **< 0.0001** |  |
| Low acclimation treatment | 0.42 | 0.17 | 2129.8 | 2.46 | **0.014** |  |
| Sub-tropical latitude group | 1.08 | 2.05 | 3 | 0.53 | 0.635 |  |
| Temperate latitude group | -3.15 | 2.05 | 3 | -1.54 | 0.220 |  |
| Rate* High acclimation treatment | -1.37 | 0.58 | 2129.8 | -2.34 | **0.019** |  |
| Rate* Low acclimation treatment | -1.42 | 0.58 | 2129.8 | -2.44 | **0.015** |  |
| Rate*Sub-tropical group | -2.38 | 0.59 | 2129.8 | -4.00 | **<0.0001** |  |
| Rate*Temperate group | -1.95 | 0.58 | 2129.8 | -3.26 | **< 0.001** |  |
| High acclimation treatment *Sub-tropical group | -0.86 | 0.24 | 2129.8 | -3.57 | **<0.001** |  |
| Low acclimation treatment *Sub-tropical group | -0.55 | 0.24 | 2129.8 | -2.28 | **0.022** |  |
| High acclimation treatment *Temperate group | 0.22 | 0.24 | 2129.8 | 0.90 | 0.367 |  |
| Low acclimation treatment *Temperate group | -0.56 | 0.24 | 2129.8 | -2.33 | **0.020** |  |
| Rate* High acclimation treatment *Sub-tropical group | 0.68 | 0.84 | 2129.8 | 0.81 | 0.418 |  |
| Rate* Low acclimation treatment *Sub-tropical group | 1.49 | 0.83 | 2129.8 | 1.79 | 0.074 |  |
| Rate* High acclimation treatment *Temperate group | -0.23 | 0.83 | 2129.8 | -0.28 | 0.780 |  |
| Rate* Low acclimation treatment *Temperate group | -1.28 | 0.82 | 2129.8 | -1.56 | 0.121 |  |
| CTmin: *lmer(CTmin~Rate*Acc*LatGrp+(1\| Fam:Gen:Sp))* |  |  |  |  |  |  |
| Intercept | -5.98 | 1.05 | 3.2 | -5.70 | **0.009** |  |
| Rate of temperature change | 3.23 | 1.14 | 1571 | 2.83 | **0.004** |  |
| High acclimation treatment | 2.17 | 0.28 | 1571 | 7.72 | **< 0.0001** |  |
| Low acclimation treatment | 0.33 | 0.27 | 1571 | 1.19 | 0.233 |  |
| Sub-tropical latitude group | 4.82 | 1.48 | 3.2 | 3.25 | **0.043** |  |
| Temperate latitude group | 4.69 | 1.48 | 3.2 | 3.16 | **0.046** |  |
| Rate* High acclimation treatment | -8.13 | 1.64 | 1571 | -4.96 | **<0.0001** |  |
| Rate* Low acclimation treatment | -2.74 | 1.62 | 1571 | -1.70 | 0.09 |  |
| Rate*Sub-tropical group | -2.73 | 1.62 | 1571 | -1.68 | 0.09 |  |
| Rate*Temperate group | -7.16 | 1.61 | 1571 | -4.44 | **<0.0001** |  |
| High acclimation treatment *Sub-tropical group | -0.71 | 0.39 | 1571 | -1.81 | 0.071 |  |
| Low acclimation treatment *Sub-tropical group | -2.18 | 0.39 | 1571 | -5.52 | **<0.0001** |  |
| High acclimation treatment *Temperate group | 1.76 | 0.39 | 1571 | 4.45 | **< 0.0001** |  |
| Low acclimation treatment *Temperate group | -0.28 | 0.39 | 1571 | -0.72 | 0.473 |  |
| Rate* High acclimation treatment *Sub-tropical group | 8.56 | 2.30 | 1571 | 3.72 | **<0.001** |  |
| Rate* Low acclimation treatment *Sub-tropical group | 1.86 | 2.32 | 1571 | 0.80 | 0.422 |  |
| Rate* High acclimation treatment *Temperate group | 4.25 | 2.30 | 1571 | 1.85 | 0.065 |  |
| Rate* Low acclimation treatment *Temperate group | 2.10 | 2.29 | 1571 | 0.92 | 0.358 |  |
| *Warming tolerance: WT~LatGrp*Rate*Treatment+(1\| Fam:Gen:Sp)* | |  |  |  |  |  |
| Intercept | 33.19 | 1.45 | 3 | 22.91 | **< 0.0001** |  |
| Sub-tropical latitude group | -26.16 | 2.05 | 3 | -12.77 | **< 0.001** |  |
| Temperate latitude group | -23.26 | 2.05 | 3 | -11.35 | **0.001** |  |
| Rate of temperature change | 9.30 | 0.42 | 2110 | 22.29 | **< 0.0001** |  |
| High acclimation treatment | 0.99 | 0.17 | 2110 | 5.83 | **< 0.001** |  |
| Low acclimation treatment | 0.39 | 0.17 | 2110 | 2.27 | **0.023** |  |
| Sub-tropical*Rate | -2.35 | 0.60 | 2110 | -3.94 | **< 0.0001** |  |
| Temperate*Rate | -1.93 | 0.58 | 2110 | -3.29 | **0.001** |  |
| Sub-tropical*High acclimation treatment | -0.87 | 0.24 | 2110 | -3.56 | **< 0.001** |  |
| Temperate*High acclimation treatment | 0.21 | 0.24 | 2110 | 0.89 | 0.376 |  |
| Sub-tropical*Low acclimation treatment | -0.52 | 0.24 | 2110 | -2.16 | **0.031** |  |
| Temperate*Low acclimation treatment | -0.53 | 0.24 | 2110 | -2.21 | **0.027** |  |
| Rate*High acclimation treatment | -1.37 | 0.59 | 2110 | -2.33 | **0.020** |  |
| Rate*Low acclimation treatment | -1.40 | 0.58 | 2110 | -2.40 | **0.017** |  |
| Sub-tropical*Rate*High acclimation treatment | 0.70 | 0.84 | 2110 | 0.83 | 0.406 |  |
| Temperate*Rate*High acclimation treatment | -0.23 | 0.83 | 2110 | -0.28 | 0.783 |  |
| Sub-tropical*Rate*Low acclimation treatment | 1.47 | 0.83 | 2110 | 1.77 | 0.078 |  |
| Temperate*Rate*Low acclimation treatment | -1.30 | 0.83 | 2110 | -1.57 | 0.116 |  |

**Table S6:** Model selection using maximum likelihood for models testing the effects of rates of temperature change (Rate), acclimation treatment (Acc; low, control and high) and latitude groups (LatGrp; polar, temperate and sub-tropical) on critical thermal maximum (CTmax), minimum (CTmin) and warming tolerance (WT). Species (Sp) was included as a random factor for the Linear Mixed Effects Models (lmer), and excluded from the Generalised Least Squares Linear Models (gls). The best fit models (in bold) were selected using the Aikaike Information Criterion (AIC) and delta AIC are presented as dAIC.

|  | Model | df | AIC | dAIC |
| --- | --- | --- | --- | --- |
| CTmax | **lmer(Ctmax~Rate*Acc*LatGrp+(1\|Sp),REML=F)** | **14** | **6502.277** | **0.0** |
|  | lmer(Ctmax~Rate+Acc*LatGrp+(1\|Sp),REML=F) | 9 | 6550.741 | 48.5 |
|  | lmer(Ctmax~Rate*Acc+LatGrp+(1\|Sp),REML=F) | 8 | 6663.031 | 160.8 |
|  | lmer(Ctmax~Rate+Acc+LatGrp+(1\|Sp),REML=F) | 7 | 6663.850 | 161.6 |
|  | gls(Ctmax~Rate*Acc*LatGrp,method="ML") | 13 | 8643.734 | 2141.5 |
| CTmin | **lmer(Ctmin~Rate*Acc*LatGrp+(1\|Sp),REML=F)** | **14** | **5496.961** | **0.0** |
|  | lmer(Ctmin~Rate+Acc*LatGrp+(1\|Sp),REML=F) | 9 | 5542.397 | 45.4 |
|  | lmer(Ctmin~Rate*Acc+LatGrp+(1\|Sp),REML=F) | 8 | 5714.870 | 217.9 |
|  | lmer(Ctmin~Rate+Acc+LatGrp+(1\|Sp),REML=F) | 7 | 5715.928 | 219.0 |
| WT | gls(Ctmin~Rate*Acc*LatGrp,method="ML") | 13 | 6201.671 | 704.7 |
|  | **lmer(WT~LatGrp*Rate*Trt+(1\|Sp),REML=F)** | **20** | **6421.382** | **0.0** |
|  | lmer(WT~LatGrp*Rate+Trt+(1\|Sp),REML=F) | 10 | 6564.931 | 143.5 |
|  | lmer(WT~LatGrp+Rate*Trt+(1\|Sp),REML=F) | 10 | 6597.529 | 176.1 |
|  | lmer(WT~LatGrp+Rate+Trt+(1\|Sp),REML=F) | 8 | 6610.934 | 189.6 |
|  | gls(WT~LatGrp*Rate*Trt,method="ML") | 19 | 8577.824 | 2156.4 |


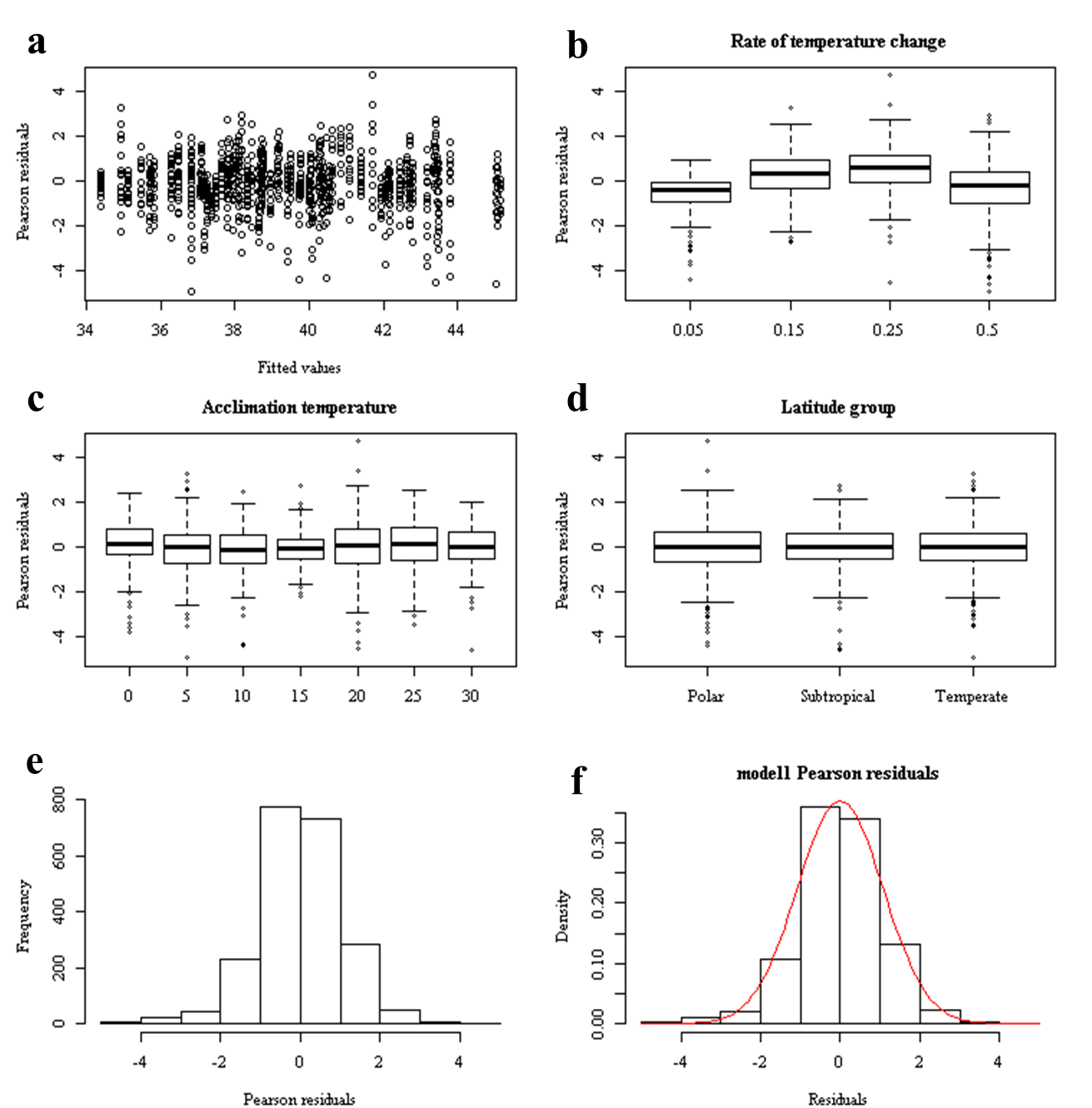


**Figure S1:** Model validation for LMM performed on CTmax of Collembola species from polar, temperate and subtropical latitude groups. Treatments are acclimation treatment and rate of temperature change. Pearson residuals from the model are plotted (a) against fitted values to assess homogeneity, (b) against rate of temperature change, (c) against acclimation temperature, and (d) against latitude group to assess independence, as well as (e) as a frequency histogram and (f) as a density histogram with normal distribution line (red line) overlaid to assess normality.


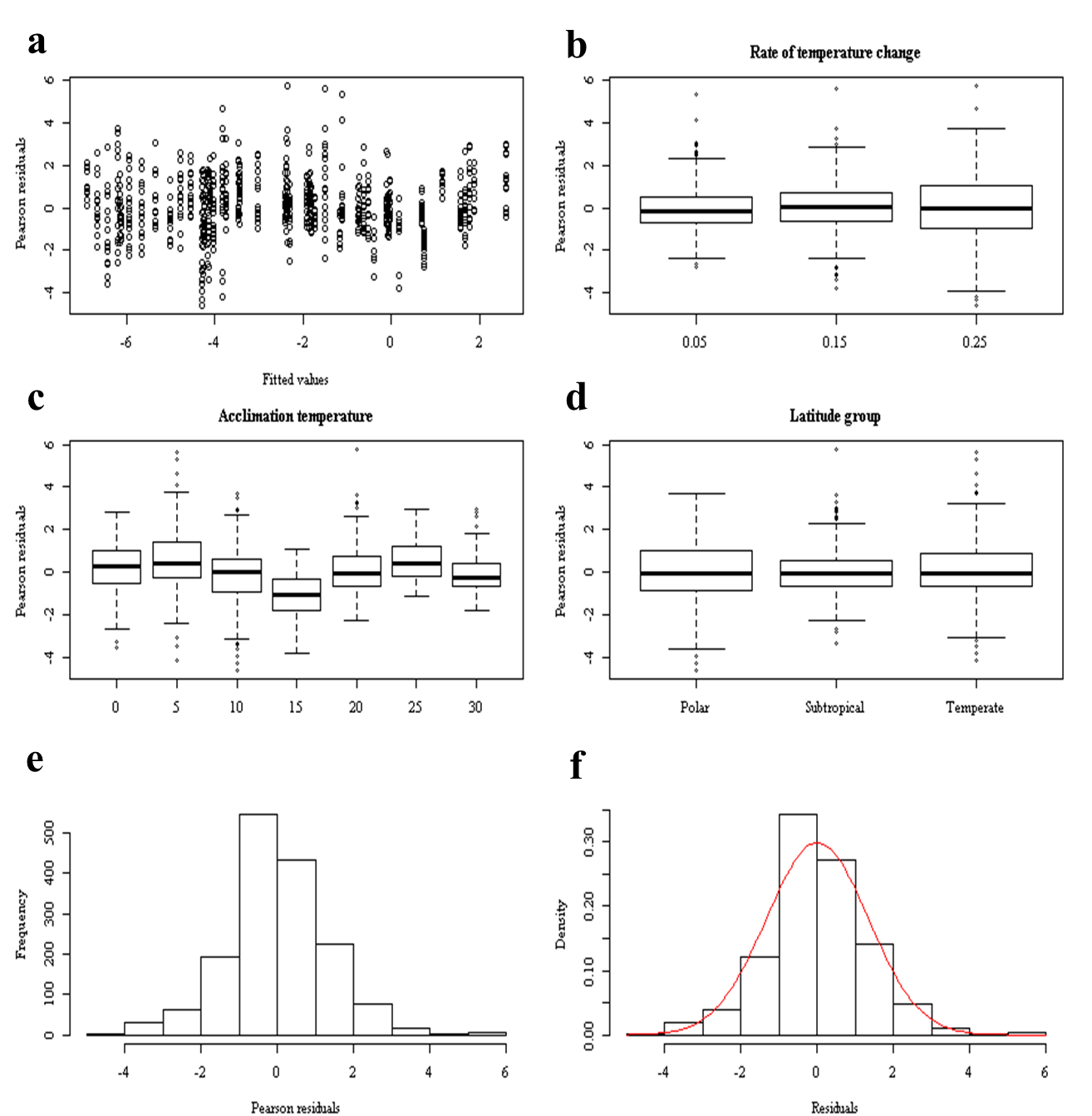


Figure S2: Model validation for LMM performed on CTmin of Collembola species from polar, temperate and subtropical latitude groups. Treatments are acclimation and rate of temperature change. Pearson residuals from the model are plotted (a) against fitted values to assess homogeneity, (b) against rate of temperature change, (c) against acclimation temperature, and (d) against latitude group to assess independence, as well as (e) as a frequency histogram and (f) as a density histogram with normal distribution line (red line) overlaid to assess normality.


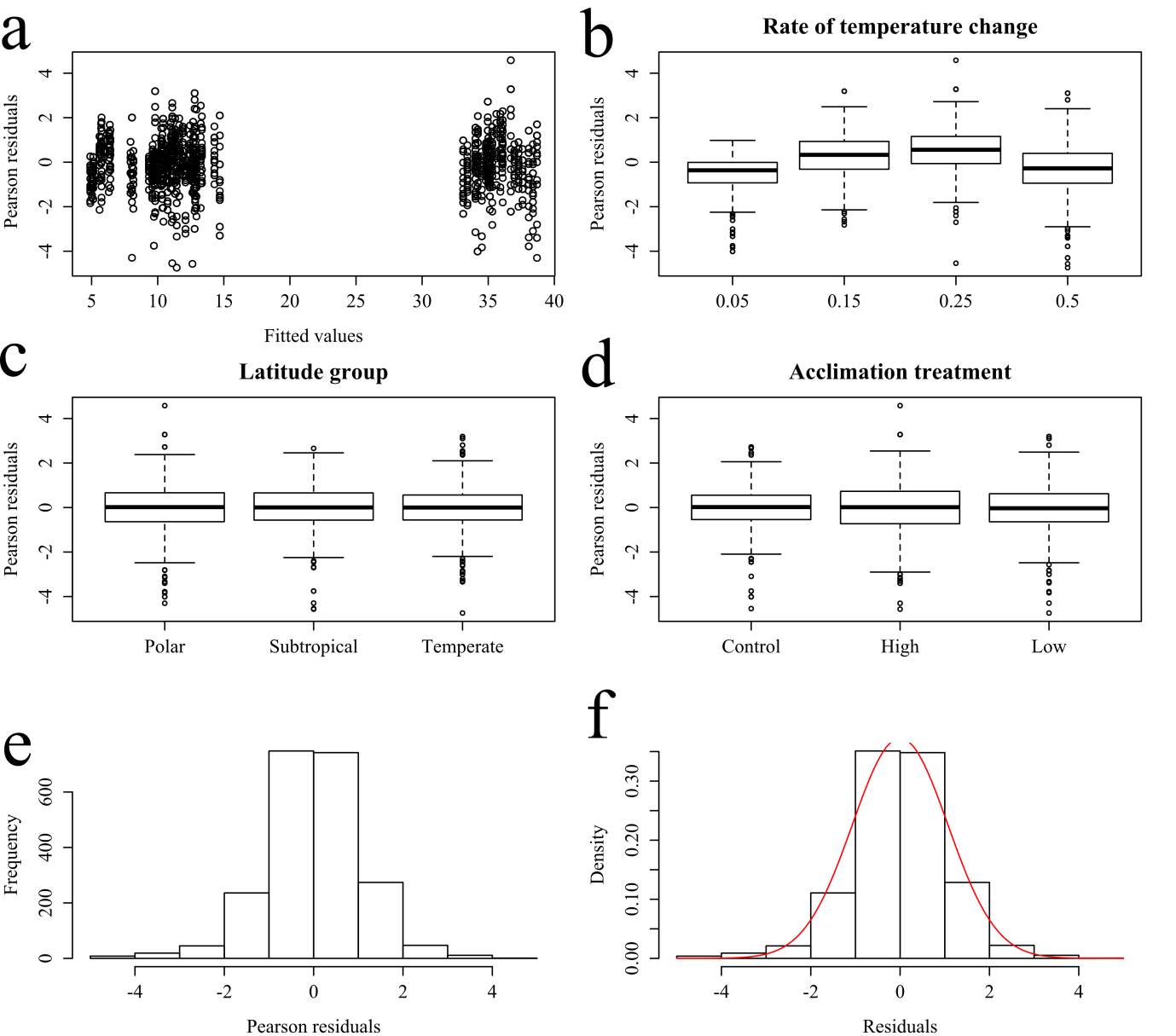


Figure S3: Model validation for LMM performed on warming tolerances of Collembola species from polar, temperate and subtropical latitude groups. Pearson residuals from the model are plotted (a) against fitted values to assess homogeneity, (b) against rate of temperature change, (c) against latitude group, (d) acclimation treatment to assess independence, as well as (e) as a frequency histogram and (f) as a density histogram with normal distribution line (red line) overlaid to assess normality.
